# [Retracted] miR‑489 promotes apoptosis and inhibits invasiveness of glioma cells by targeting PAK5/RAF1 signaling pathways

**DOI:** 10.3892/or.2023.8564

**Published:** 2023-05-08

**Authors:** Wei Wang, Luyang Zhang, Wei Gao, Dongyong Zhang, Zilong Zhao, Yijun Bao

Oncol Rep 42: 2390–2401, 2019; DOI: 10.3892/or.2019.7381

Subsequently to the publication of the article, an interested reader drew to the authors' attention that certain of the data panels showing the results of cell migration and invasion assays in Figs. 5A and 6C were overlapping, suggesting that the data were derived from the same original source, even though they were selected to represent the results from differently performed experiments.

The authors requested that a corrigendum be published to rectify this problem; however, after having conducted an independent analysis of the data in the Editorial Office, we have noticed that the data shown in Figs. 5A and 6C are strikingly similar to data appearing in different form in other articles published in another journal, mainly written by different authors at different research institutions. Owing to the fact that the contentious data in the above article were already under consideration for publication, or had already been published, elsewhere at the time it was submitted to *Oncology Reports*, the Editor has decided that this paper should be retracted from the Journal. After having been in contact with the authors, they agreed with the decision to retract the paper. The Editor apologizes to the readership for any inconvenience caused.

